# Velocity-based resistance training: do women need greater velocity loss to maximize adaptations?

**DOI:** 10.1007/s00421-022-04925-3

**Published:** 2022-03-08

**Authors:** J. Rissanen, S. Walker, F. Pareja-Blanco, K. Häkkinen

**Affiliations:** 1 NeuroMuscular Research Center, Faculty of Sport and Health Sciences, University of Jyväskylä, Room VIV225, 40014 Jyväskylä, Finland; 2 Department of Sports and Computer Sciences, Physical Performance and Sports Research Center, Universidad Pablo de Olavide, Seville, Spain

**Keywords:** Strength training, Training volume, Sex differences, Muscle activity, Mean propulsive velocity, Hypertrophy

## Abstract

**Purpose:**

Men and women typically display different neuromuscular characteristics, force–velocity relationships, and differing strength deficit (upper vs. lower body). Thus, it is not clear how previous recommendations for training with velocity-loss resistance training based on data in men will apply to women. This study examined the inter-sex differences in neuromuscular adaptations using 20% and 40% velocity-loss protocols in back squat and bench press exercises.

**Methods:**

The present study employed an 8-week intervention (2 × week) comparing 20% vs. 40% velocity-loss resistance training in the back squat and bench press exercises in young men and women (~ 26 years). Maximum strength (1-RM) and submaximal-load mean propulsive velocity (MPV) for low- and high-velocity lifts in squat and bench press, countermovement jump and vastus lateralis cross-sectional area were measured at pre-, mid-, and post-training. Surface EMG of quadriceps measured muscle activity during performance tests.

**Results:**

All groups increased 1-RM strength in squat and bench press exercises, as well as MPV using submaximal loads and countermovement jump height (*P* < 0.05). No statistically significant between-group differences were observed, but higher magnitudes following 40% velocity loss in 1-RM (*g* = 0.60) and in low- (*g* = 1.42) and high-velocity (*g* = 0.98) lifts occurred in women. Training-induced improvements were accompanied by increases in surface EMG amplitude and vastus lateralis cross-sectional area.

**Conclusion:**

Similar increases in strength and power performance were observed in men and women over 8 weeks of velocity-based resistance training. However, some results suggest that strength and power gains favor using 40% rather than 20% velocity loss in women.

## Introduction

The percentage of one-repetition maximum (% of 1-RM) has been traditionally used to determine loading intensity during specific resistance training programming (e.g., 0–60% 1-RM for power training) (American College of Sports Medicine 2009). This requires a 1-RM test in the specific exercise to be performed regularly throughout the training cycle. Such practice may not be optimal given that 1-RM testing: (1) requires a non-fatigued condition before the test, thus, affecting training session timetabling, (2) is itself highly fatiguing, and (3) may affect performance in subsequent training sessions. Furthermore, 1-RM performance changes daily (1.1‒17.5 kg in lower-body and 0.5‒4.9 kg in upper-body exercises) (Grgic et al. 2020). Such factors may make precise load prescription difficult, even with 1-RM testing.

Another important variable in dosing resistance training for individuals is the number of repetitions per set. Traditionally, pre-determined repetition ranges have been used for this purpose (e.g., 1–6 repetitions per set for power training) (American College of Sports Medicine 2009). Nevertheless, different individuals can perform a distinct number of repetitions per set with the same relative load (González-Badillo et al. 2017) likely due to their inherent neuromuscular characteristics, such as capillary density (Terzis et al. 2008) and/or muscle fiber composition (Douris et al. 2006). Therefore, the produced training stimuli from generic resistance training programs may be very different for each individual; i.e., some receiving too little (volume) stimulus and some receiving sufficient or even too great (volume) stimulus.

Velocity-based resistance training (VBRT) is a resistance training method where movement velocity is used to determine both intensity and volume dosage. VBRT requires that the concentric phase of the exercise is performed with maximum velocity and the velocity of each individual repetition is measured. VBRT can be used to estimate the daily 1-RM using the concentric velocity of the first repetition of the exercise (after warm-up sets) to adjust the training load for that training session (González-Badillo and Sánchez-Medina 2010; Sánchez-Medina et al. 2017). Moreover, velocity loss during each set can be used to terminate the set and, in doing so, target specific training adaptations (González-Badillo et al. 2017; Pareja-Blanco et al. 2017). For example, Pareja-Blanco et al. (2017) showed that higher volume (40% velocity loss) is more beneficial for muscle hypertrophy but a clear shift towards a slower phenotype (from type IIX to type IIA muscle fibers) occurred. Conversely, a lower training volume (20% velocity loss) potentially provides greater benefit for countermovement jump (CMJ), i.e., power performance, in men.

The first scientific publication investigating VBRT was published in 2010. Thereafter, over 70 scientific articles have been published on the topic, in which > 50 have been published in the last two years. Nevertheless, women have been much less studied within this topic; only 16 in the last five years, with most being cross-sectional comparisons. This is an important gap in the literature given the typical differences in neuromuscular characteristics and in acute neuromuscular fatigue response to a single resistance training session between sexes (Ansdell et al. 2019; Linnamo et al. 1998; Häkkinen 1994). As the type and magnitude of acute fatigue is important to the overall adaptation to training (Ahtiainen et al. 2003; Pareja-Blanco et al. 2017; Walker et al. 2013), it could be hypothesized that VBRT observations previously described in men are not necessarily directly applicable to women. From both scientific and practical perspectives, it is important to determine the effects of different velocity-loss protocols on neuromuscular adaptations between men and women.

In addition, a limited number of studies have been conducted on the adaptations between upper- and lower-body exercises in the same intervention; where women’s strength deficit compared to men is greater in the upper- than the lower-limbs (Miller et al. 1993). Men and women have also shown different load–velocity relationships in upper- versus lower-body exercises (Askow et al. 2019; Pareja-Blanco et al. 2020a, b, c; Torrejón et al. 2019). Combining these considerations with the already known between-sex differences in neuromuscular characteristics and fatiguability (Ansdell et al. 2019; Linnamo et al. 1998; Häkkinen 1994), we hypothesized that neuromuscular adaptations might differ between sexes and/or between upper- and lower-body exercises when using the same training programming in VBRT. The present study investigated inter-sex differences in neuromuscular adaptations using 20% and 40% velocity-loss protocols in back squat and bench press exercises over 8 weeks of training.

## Materials and methods

### Subjects

Twenty-four healthy young men (26.4 ± 3.9 years, 181.1 ± 5.4 cm, 81.9 ± 11.3 kg) and twenty-five women (25.5 ± 3.8 years, 166.6 ± 7.2 cm, 60.7 ± 6.1 kg) were pair-matched to four different training groups: men training until 20% velocity loss (VL20M, *n* = 12) or 40% velocity loss (VL40M, *n* = 12), and women training until 20% velocity loss (VL20W, *n* = 13) or 40% velocity loss (VL40W, *n* = 12) within each set. Subjects were allocated based upon their sum of 1-RM in back squat and bench press following a reverse counterbalancing (i.e., ABBA) sequence, after separation of men and women. All subjects were motivated, free of any illness or injuries and had at least one-year experience of systematic resistance training with the back squat and bench press exercises commonly used as part of their recreational training program. Before inclusion, the subject underwent a medical evaluation process, which included resting ECG scan and medical history questionnaire that were examined by a physician. All included subjects signed informed (written and orally) consent forms, and the study was approved by the local ethics committee.

During the intervention, 3 women (two from VL20W and one from VL40W) dropped out because of injuries (not obtained during the study) or personal reasons. In addition, one man (in VL40M) was not able to complete the squat training program due to injury sustained during the study’s training intervention, although he completed bench press training and testing. The final number of subjects in each group was: VL20M: *n* = 12, VL40M: *n* = 11, VL20W: *n* = 11, VL40W: *n* = 11 and characteristics upon entering the study are presented in Table 1. Subjects were requested to not perform any other type of strenuous physical activity during the study period.

**Table 1:** Baseline characteristics of the groups

Group	*n*	Height (cm)	Body mass (kg)	Control SQ 1-RM (kg)	Control BP 1-RM (kg)
VL20M	12	183.6 ± 7.9	81.5 ± 8.0	112.3 ± 28.2	80.6 ± 17.4
VL40M	11	178.7 ± 5.9	82.3 ± 14.3	111.2 ± 14.0	78.3 ± 13.6
VL20W	11	167.0 ± 6.8	61.2 ± 4.6	67.8 ± 12.3	40.2 ± 9.9
VL40W	11	165.1 ± 7.1	60.1 ± 7.5	66.0 ± 18.8	39.3 ± 9.9

VL20M = men training until 20% of velocity loss, VL40M = men training until 40% of velocity loss, VL20W = women training until 20% of velocity loss, VL40W = women training until 40% of velocity loss within each set. SQ = back squat exercise; BP = bench press exercise; 1-RM = one-repetition maximum

### Experimental design

The present study consisted of an 8-week VBRT intervention in the squat and bench press exercises. Two groups, VL20M and VL20W, performed their sets during the training program until they reached a velocity loss of 20%. The other two groups, VL40M and VL40W, performed their sets until they achieved a velocity loss of 40%. All groups completed a total of 15 training sessions over the 8-week training period (Table 2). The subjects trained twice per week in weeks 1–4 and 6–8, with one training session held in week 9 prior to post-tests. Mid-training tests were performed in week 5 and no training sessions took place. The subjects were tested on four occasions during the study: Control-tests (Week 2, Control), before training (Week 0, PRE), after 8 training sessions (Week 5, MID) and after 15 training sessions (Week 10, POST). The measurements were conducted on two different sessions separated by < 48 h. The first testing session consisted of body mass and muscle size measurements. The second testing session consisted of CMJ and progressive loading tests in the Smith-machine full back squat (SQ) and the Smith-machine bench press (BP) exercises, in that order. Both testing and training sessions were performed in a research laboratory under the direct supervision of investigators, at the same time of the day (± 1 h) and under the same environmental conditions.

**Table 2:** Descriptive characteristics of the 8-week velocity-based back squat and bench press training program performed by the four experimental groups

	Session 1	Session 2	Session 3	Session 4	Session 5	Session 6	Session 7	Session 8
Set x %1-RM	2 × 65	3 × 65	3 × 65	4 × 65	5 × 65	3 × 70	3 × 70	4 × 70

	Mid-test	Session 9	Session 10	Session 11	Session 12	Session 13	Session 14	Session 15
Set x %1-RM		5 × 70	5 × 70	3 × 75	4 × 75	4 × 75	5 × 75	5 × 75

	Fastest MPV (m s^−1^)	Slowest MPV (m s^−1^)	MPV all reps	Mean VL	Total rep
Back squat					
VL20M	0.79 ± 0.06	0.59 ± 0.04^40M, 40W^	0.73 ± 0.02^40M, 40W^	22.1 ± 1.0^40M, 40W^	278.7 ± 70.3^40W^
VL40M	0.81 ± 0.04	0.47 ± 0.03	0.63 ± 0.02	41.7 ± 1.5	397.1 ± 98.0
VL20W	0.80 ± 0.05	0.62 ± 0.03^40M, 40W^	0.73 ± 0.02^40M, 40W^	21.5 ± 1.0^40M, 40W^	373.9 ± 135.4^40W^
VL40W	0.80 ± 0.04	0.45 ± 0.02	0.64 ± 0.03	40.5 ± 0.7	518.6 ± 137.1
Bench press					
VL20M	0.64 ± 0.02	0.46 ± 0.02^40M, 40W^	0.56 ± 0.01^40M, 40W^	22.6 ± 0.6^40M, 40W^	258.6 ± 59.5^40M, 40W^
VL40M	0.62 ± 0.03	0.33 ± 0.02	0.48 ± 0.02^40W^	42.4 ± 1.3	413.0 ± 67.2
VL20W	0.62 ± 0.04	0.45 ± 0.04^40M, 40W^	0.56 ± 0.02^40M, 40W^	22.3 ± 0.9^40M, 40W^	283.3 ± 54.3^40M, 40W^
VL40W	0.61 ± 0.03	0.34 ± 0.02	0.50 ± 0.01	41.4 ± 0.9	491.0 ± 113.8

Data are mean ± SD. VL20M: Men that trained with a mean velocity loss of 20% in each set (*n* = 12); VL40M: Men that trained with a mean velocity loss of 40% in each set (*n* = 11); VL20W: Women that trained with a mean velocity loss of 20% in each set (*n* = 10); VL40W: Women that trained with a mean velocity loss of 40% in each set (*n* = 11). MPV: Mean Propulsive Velocity; Fastest MPV: Average of the fastest repetitions measured in each session; Slowest MPV: Average of the slowest repetitions measured in each session; MPV all reps: Average MPV attained during the entire training program (excluding the warm-up repetitions); Mean VL: Average velocity loss attained during the entire training program; Total rep: Total number of repetitions performed during the training program (excluding the warm-up repetitions). Statistically significant differences versus VL40M: ^40M^*P* ≤ 0.05. Statistically significant differences versus VL40W: ^40W^*P* ≤ 0.05

### Measurements

*Smith-machine full back squat incremental loading test*: The subjects were instructed to perform the eccentric phase of the exercise descending at moderate velocity (approx. 2 s decent) until the thighs were bellow parallel, while the concentric phase was performed with maximal velocity. The feet needed to stay in contact with the ground and the bar on the shoulders during the whole exercise. MPV of each testing load was recorded using a linear velocity transducer and software (T-Force System, Ergotech, Murcia, Spain). The initial load was 41 kg in men and 31 kg in women. The load was increased with 10‒15 kg steps in each set until MPV of the trial was lower than 0.5 m s^−1^. Thereafter, the load was increased by 5 or 2.5 kg in each set. Subjects were allowed to perform from one (in the heaviest sets) to six (in the lightest sets) repetitions per measured load. Recovery periods between sets were 2 min until the MPV was 0.5 m s^−1^ and were 3 min thereafter. Strong verbal encouragement was used by a researcher during the concentric phase of the repetition. The measurement ended when the subject reached the load that led to failure. The same absolute incremental loads were tested in Control, PRE, MID and POST to be able to compare the MPV change between the tests. If the subject successfully achieved their previous 1-RM, the test was continued until failure.

MPV performances were retrospectively classified as “low” or “high” velocity according to our previously published sex-specific values (Pareja-Blanco et al. 2020c). The cut-off was set at 70% 1-RM for both exercises, which was the median relative load within the range used during training (65–75%1-RM, Table 2). In SQ, cut-offs of 0.73 and 0.65 m s^−1^ were used for men and women, respectively. The highest MPV score from each absolute load was taken as the best performance, and then mean MPV from all low and all high velocities were calculated for each subject. The coefficient of variation (CV) for Control to PRE was 2.8% for 1-RM, 5.6% for MPV with low velocities and 14.0% for MPV with high velocities.

*Smith-machine bench press incremental loading test*: MPV of each repetition during the test was recorded using the same measurement system as in the SQ incremental loading test (T-Force System, Ergotech, Murcia, Spain). The initial load was 36 kg in men and 10–20 kg in women depending on the estimated 1-RM of the individual subject. The load was increased in 2.5‒15 kg steps in each set until MPV of the trial slowed to 0.4 m s^−1^. Thereafter, the load was increased by 5 or 2.5 kg in men and by 2.5 or 1 kg in women. The subjects were given instructions to perform from one (in the heaviest sets) to six (in the lightest sets) repetitions per set in this exercise, as in SQ. The recovery periods between sets were 2 min when the highest MPV in the last set was over 0.4 m s^−1^ and 3 min when MPV in the last set was under 0.4 m s^−1^. The subjects were advised to perform the eccentric phase of the exercise using moderate velocity and stop the bar on the chest. After a ~ one-second pause, a verbal signal was given by a researcher and the subjects performed the concentric phase of the movement as fast as possible. The stop on the chest was used to minimize bouncing on the chest and, thus, to standardize the technique (Pallarés et al. 2014). The bar needed to stay in the hands of the subject during the whole exercise. Strong verbal encouragement was used by a researcher during the concentric phase of the repetition. The measurement ended when the subject reached the load that led to failure. If the subject achieved their previous 1-RM, the test continued until failure. The cut-off to distinguish between “low” and “high” velocity, corresponding to 70% 1-RM, were 0.58 (men) and 0.54 (women) m s^−1^ (Pareja-Blanco et al. 2020c). The CV for Control to PRE was 2.7% for 1-RM, 4.5% for MPV with low velocities and 10.6% for MPV with high velocities.

*Countermovement jumps (CMJ)* were performed on a force platform (Faculty of Sport and Health Sciences at the University of Jyväskylä, Finland) with Signal software version 4.14 (Cambridge Electronic Design Ltd., Cambridge, United Kingdom) used to record (sampling rate 2000 Hz) and analyze data. Vertical displacement of a subject’s center of mass was calculated using the following equation: *h* = *I*^2^/(2 gm^2^), where *h* = vertical displacement of a subject’s center of mass, *I* = vertical impulse of force, *g* = acceleration due to gravity, *m* = mass of the subject. The subjects performed the measurement by lowering their body rapidly and then pushing against the ground with an explosive change of direction. Hands were kept on the hips throughout the whole movement and the subjects were encouraged to jump as high as they can in every measurement. Subjects performed 3 jumps separated by 1 min of rest, and the highest of the 3 jumps was taken into further analyses. The CV for Control to PRE was 3.8%.

*Surface electromyography (EMG)* was recorded from the vastus lateralis and medialis of the right leg during SQ and CMJ. Bipolar silver-silver chloride surface electrodes (Ambu BlueSensor N, Copenhagen, Denmark) with an inter-electrode distance of 20 mm were attached to muscle-specific locations following SENIAM guidelines (Hermens et al. 2000). Small indelible ink tattoos (diameter < 1.0 mm) were placed during Control to ensure that the electrodes were replaced exactly to the same spots in each measurement (Häkkinen and Komi 1983). The telemetric recording system (Noraxon Inc. Scottsdale, Arizona, USA) with a sampling frequency of 3000 Hz was used for data collection. The EMG signal was down-sampled to 2000 Hz and transmitted via A/D converter (Cambridge Electronic Design Ltd., Cambridge, United Kingdom) to a personal computer where Signal 4.14 software (Cambridge Electronic Design Ltd., Cambridge, United Kingdom) was used to data recording and analyzing. The data were band-pass filtered over 20–350 Hz and root-mean squared for both muscles separately, and the average of vastus lateralis and medialis EMG activity (lateralis + medialis/2) were analyzed over the entire concentric phase of the CMJ measurement and back squat 1-RM trial.

*Muscle size* was measured by anatomical cross-sectional area (CSA) in the axial plane of vastus lateralis and by muscle thickness of the triceps brachii, both from the right limbs, using a B-mode ultrasound device (SSD-a10; Aloka, Tokyo, Japan). A 10 MHz linear-array probe (60 mm) coated with water-soluble transmission gel and housed in a custom-made convex support was used. CSA measurements were performed using the extended-field-of-view function as previously described (Walker and Häkkinen 2014; Walker et al. 2020), which has been shown to be a valid and repeatable method when assessing muscle CSA changes over time (Ahtiainen et al. 2010). Triceps brachii measurements were taken at the mid-point between the medial epicondyle and the acromion. The probe was oriented in perpendicular to the skin and positioned with minimal contact to avoid tissue deformation. Three images were taken during each measurement and the average of the three values was taken forward for further analyses. All measurements were taken and analyzed (ImageJ software version 1.44, National Institutes of Health, Bethesda, Maryland, USA) by the same experienced researcher. The CV for Control to PRE was 3.5% for vastus lateralis CSA and 4.0% for triceps brachii thickness.

*Body mass* was measured between 7 and 8 am. of the testing day after > 10 h of fasting using Inbody 720 scale and bioelectrical impedance device (Biospace Co., Seoul, South Korea).

### Training program

The descriptive characteristics of the training program are presented in Table 2. The training program included two main exercises, SQ and BP, which were always performed in that order. In addition, three sets of supplementary trunk exercises (8–12 reps with 1 min inter-set recovery), for abdominals and lower back, were performed at the end of each training session using low loads and moderate velocities (approx. 2 s concentric and 2 s eccentric duration). The technique for SQ and BP was as described in the Measurements section, i.e., concentric phase performed with maximum voluntary velocity, SQ performed without a pause from eccentric to concentric transition, the bar stopped on the chest ~ 1 s before the concentric phase in the BP exercise etc. The MPV of each repetition in every training session was measured with a linear velocity transducer (T-Force Measurement System, Ergotech, Murcia, Spain). In SQ and BP, the training intensity was determined by using the velocity-based estimation of 1-RM (Sánchez-Medina et al. 2017; González-Badillo and Sánchez-Medina 2010). The load of the bar was adjusted if needed after the first repetition of the first set in every training session to match with the velocity (± 0.03 m s^−1^) that corresponds the desired load (% of predicted 1-RM). The training loads were progressively increased from 65 to 75% predicted 1-RM during the training program (Table 2). The inter-set rest interval was 3 min in all sessions. Strong verbal encouragement was used in every training session to perform the concentric phases with the maximum possible velocity. Each subject performed an individual number of repetitions in each training session based on their velocity-loss allocation and their ability to withstand fatigue.

### Statistical analyses

Based on a previous study (Pareja-Blanco et al. 2020a), sample size estimations suggested that 12 subjects per group would satisfy alpha (0.05) and power (0.95) assumptions. Test–retest (Control to PRE) reliability was measured by the standard error of measurement (SEM), which was expressed in relative terms through the CV. SEM was calculated as the root mean square of the intra-subject total mean square. Mean values and standard deviations of each group were calculated according to standard procedures. All data were checked for normality using the Shapiro–Wilk test prior to performing statistical analyses. Independent *T*-tests assessed potential between-group differences between 20 and 40% velocity-loss groups (within sex) at baseline (PRE). Training session data, i.e., repetition number and velocity, were assessed by two-way ANOVA (2 sex × 2 velocity loss). Repeated measures ANCOVA (2 sex × 2 velocity loss × 4 time-points), using pre-training values as covariate, was used to evaluate the effects of the training program between sexes and velocity-loss protocols. Where appropriate, Bonferroni post hoc tests were applied for pairwise comparisons. Possible relationships between training-induced changes in tested variables was explored by Pearson’s product-moment correlation test. To examine the magnitude of differences between velocity-loss protocols (within sex), Hedge’s *g* effect sizes were calculated from between-group differences in the relative changes (Δ%) from PRE to POST. The cut-offs for small, medium and large effect were < 0.3, 0.3‒0.8, and > 0.8, respectively. All statistical analyses were performed using IBM SPSS Statistics software (SPSS version 26, Chicago, Illinois, USA).

## Results

Significant main effects for Time showed that all variables increased over the training program (*F* = 16.3‒118.5, *P* < 0.001), with the exception of triceps brachii thickness. Nevertheless, the only significant between-group effects from ANCOVA analyses were observed in CMJ height for time × sex (*F* = 5.2, *P* = 0.028) and vastus lateralis CSA for time × sex × velocity loss (*F* = 5.89, *P* = 0.020). There were also trends in low-velocity BP (MPV) for time × sex (*F* = 2.62, *P* = 0.082), time × velocity loss (*F* = 3.12, *P* = 0.053) and time × sex × velocity loss (*F* = 2.54, *P* = 0.088), and CMJ EMG for time × sex × velocity loss (*F* = 3.46, *P* = 0.070).

### Repetitions and velocities performed during training

**Fig. 1:**
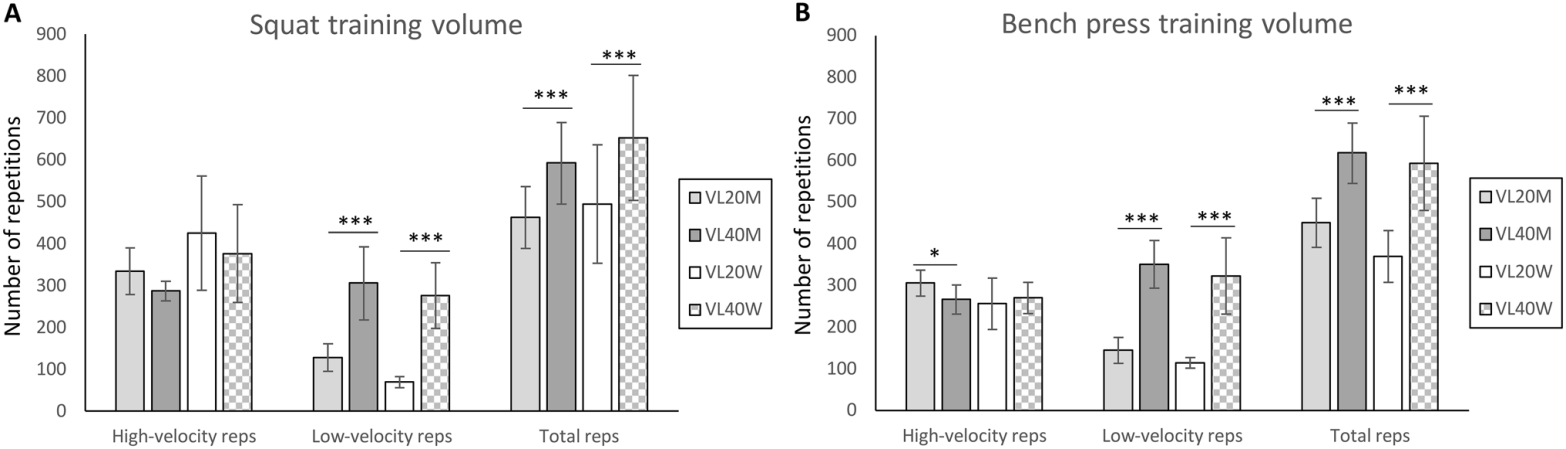
Total number of repetitions performed during the 15 training sessions in back squat (**A**) and bench press (**B**) exercises, as well as the total number of repetitions performed above (high-velocity reps) and below (low-velocity reps) the velocity corresponding to 70% of 1-RM for each sex and exercise (Pareja-Blanco et al. 2020c). Data are expressed as mean ± SD. * = *P* < 0.05 between velocity-loss protocols (within sex), *** = *P* < 0.001 between velocity-loss protocols (within sex)

Adherence to training was 98 ± 3% in VL20M, 97 ± 5% in VL40M, 95 ± 6% in VL20W, and 95 ± 4% in VL40W, with no statistical differences between-groups. The total number of repetitions completed by the 20% velocity-loss groups was ~ 78% and ~ 76% in SQ in men and women, respectively, and was ~ 73% and ~ 62% in BP in men and women, respectively, compared to their respective 40% velocity-loss groups (*P* < 0.001, Fig. 1). There was a significantly greater number of low-velocity repetitions in both 40% velocity-loss groups (*P* < 0.001). In SQ, all women (i.e., VL20W + VL40W) performed more repetitions with high velocity than all men (i.e., VL20M + VL40M) (*P* = 0.003), whereas men performed more low-velocity repetitions than women (*P* = 0.02). In BP, VL20M completed more repetitions at high velocity than VL40M (*P* = 0.03), but this difference was not observed between women (VL20W vs. VL40W). Also, all men (i.e., VL20M + VL40M) performed more repetitions than all women (i.e., VL20M + VL40M) (*P* = 0.03).

### Smith-machine full back squat 1-RM

**Fig. 2:**
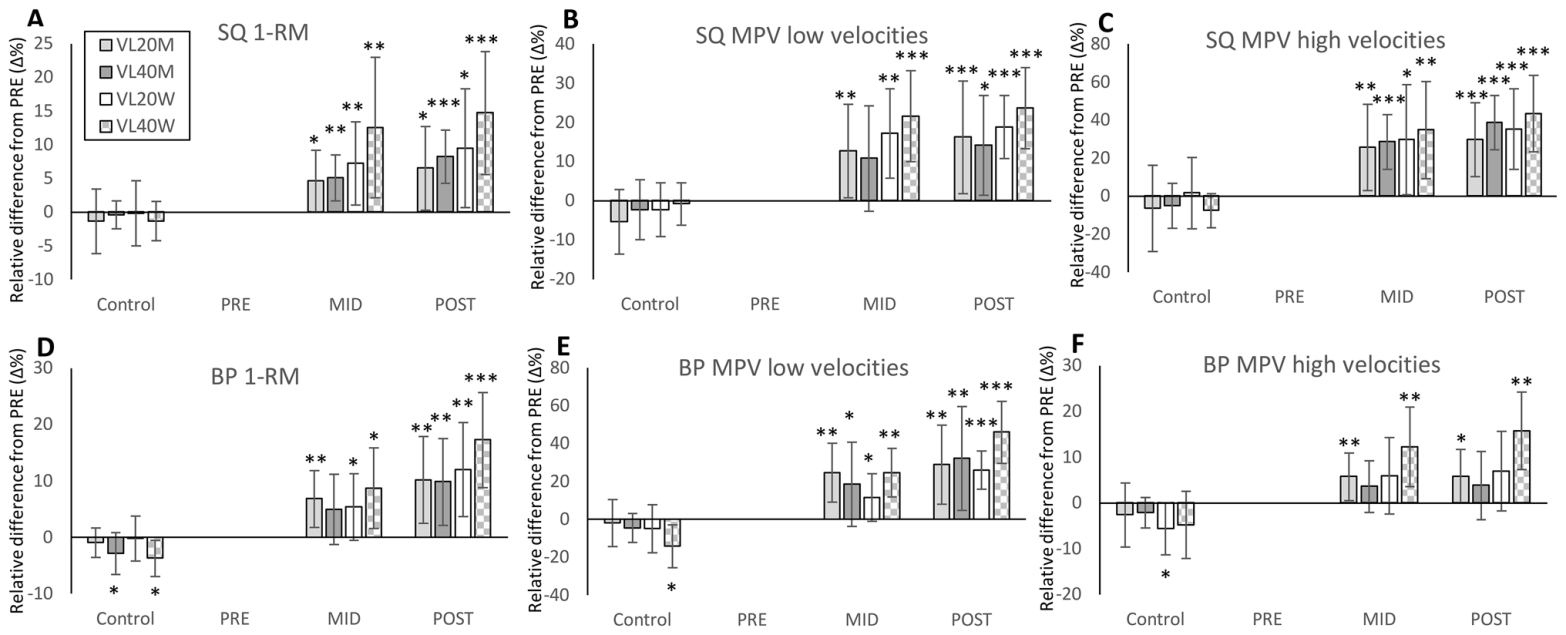
Mean (± SD) relative changes (Δ%) in back squat (SQ) and bench press (BP) for 1-RM (**A** and **D**), and mean propulsive velocity (MPV) with low velocities (**B** and **E**) and high velocities (**C** and **F**) in all groups during the 8-week velocity-based intervention. Cut-offs were 0.73 and 0.65 m s^−1^ in SQ and 0.58 and 0.54 m s^−1^ in BP for men and women, respectively (corresponding to 70% 1-RM for each sex and exercise; Pareja-Blanco et al. 2020c). VL20M = men training until 20% of velocity loss, VL40M = men training until 40% of velocity loss, VL20W = women training until 20% of velocity loss, VL40W = women training until 40% of velocity loss within each set. Within-group change compared to PRE: *P < 0.05, **P < 0.01, ***P < 0.001

At PRE, SQ 1-RM did not differ (*P* > 0.05) between VL20M (113.7 ± 28.0 kg) and VLM40 (111.7 ± 13.7 kg) nor between VL20W (68.0 ± 12.6 kg) and VL40W (67.1 ± 19.9 kg). All groups of men and women significantly increased their SQ 1-RM from PRE to MID and from PRE to POST (Fig. 2A), without significant between-group differences. Effect sizes for between-group differences in relative changes PRE to POST showed a medium effect in men (*g* = 0.31) and women (*g* = 0.56).

Surface EMG during the SQ 1-RM did not change from PRE to POST in any of the groups. Averaged EMG activity was 0.452 ± 0.179 mV (VL20M), 0.564 ± 0.144 mV (VL40M), 0.532 ± 0.135 mV (VL20W), and 0.503 ± 0.230 mV (VL40W) at PRE for the four groups.

### Smith-machine full back squat MPV

At Pre, SQ MPV did not differ (*P* > 0.05) either in high velocities or low velocities between VL20W (0.81 ± 0.08 and 0.46 ± 0.07 m s^−1^) and VL40W (0.82 ± 0.07 and 0.45 ± 0.06 m s^−1^). In men, there was a statistically significant between-group difference for low velocities (0.52 ± 0.05 vs. 0.46 ± 0.04 m s^−1^, *P* = 0.007) but not when comparing high velocities (0.97 ± 0.08 vs. 0.95 ± 0.06 m s^−1^). All groups showed statistically significant increases in MPV of both high and low velocities from PRE to POST (Fig. 2B and C), without significant between-group differences.

Effect sizes for between-group differences in relative changes PRE to POST showed a small (*g* = 0.14) and medium effect (*g* = 0.51) in men and a medium (*g* = 0.50) and medium effect (*g* = 0.38) in women for high and low velocities, respectively. Further, the training-induced changes in SQ 1-RM and MPV in high (*r* = 0.467, *P* = 0.033, *n* = 21) and low velocities (*r* = 0.471, *P* = 0.027, *n* = 22) were significantly related in women. In men, training-induced changes in SQ 1-RM and MPV at low velocities were significantly related (*r* = 0.479, *P* = 0.021, *n* = 23), but changes in 1-RM and MPV at high velocities were not (*r* = 0.194, *P* = 0.381, *n* = 23).

### Smith-machine bench press 1-RM

At PRE, BP 1-RM did not differ (P > 0.05) between VL20M (81.2 ± 16.8 kg) and VLM40 (80.5 ± 13.5 kg) nor between VL20W (40.2 ± 9.9 kg) and VL40W (39.3 ± 9.9 kg). All groups showed statistically significant increases from PRE to POST (Fig. 2D), without significant between-group differences. Effect sizes for between-group differences in relative changes PRE to POST showed a small effect (*g* = 0.04) in men but a medium effect (*g* = 0.60) in women.

### Smith-machine bench press MPV

At PRE, BP MPV did not differ (*P* > 0.05) either in high velocities or low velocities between VL20M (0.83 ± 0.09 and 0.35 ± 0.07 m s^−1^) and VL40M (0.79 ± 0.09 and 0.35 ± 0.05 m s^−1^). In women, there was a statistically significant between-group difference for low velocities (0.35 ± 0.03 m s^−1^ vs. 0.30 ± 0.06 m s^−1^, *P* = 0.039) but not for high velocities (0.71 ± 0.10 vs. 0.65 ± 0.05 m s^−1^). Both low-velocity and high-velocity MPV increased in all groups, but there were no statistically significant between-group differences in training-induced increases in MPV for low or high velocities (Fig. 2E and F).

Effect sizes for between-group differences in relative changes PRE to POST showed a small effect (*g* = 0.13) and small effect (*g* = 0.29) in men for low and high velocity, respectively. However, large between-group effect sizes in women were observed for low (*g* = 1.42) and high velocities (*g* = 0.98). As in SQ, the training-induced changes in BP 1-RM and low (*r* = 0.503, *P* = 0.024, *n* = 20) and high velocities (*r* = 0.518, *P* = 0.019, *n* = 20) were significantly related in women. In men, training-induced changes in BP 1-RM and low (*r* = 0.521, *P* = 0.011, *n* = 23) and high velocities (*r* = 0.744, *P* < 0.001, *n* = 23) were also significantly related.

### Countermovement jump

**Fig. 3:**
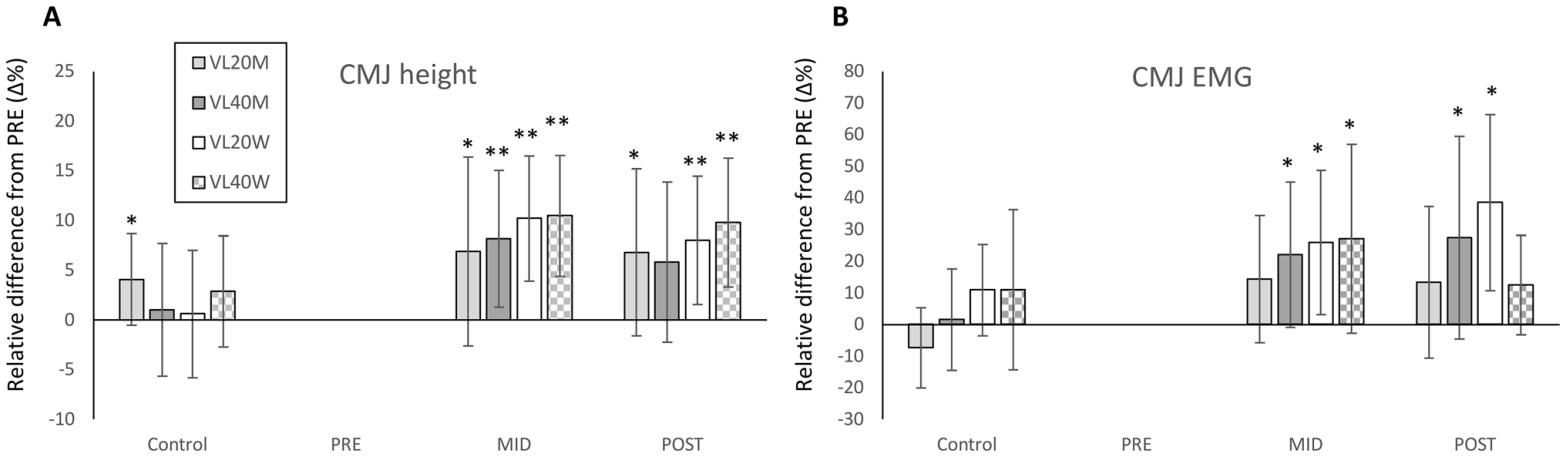
Mean (± SD) relative changes (Δ%) in countermovement jump (CMJ) height (**A**) and averaged EMG during the concentric phase of the CMJ (**B**) in all groups during the 8-week velocity-based intervention. VL20M = men training until 20% of velocity loss, VL40M = men training until 40% of velocity loss, VL20W = women training until 20% of velocity loss, VL40W = women training until 40% of velocity loss within each set. Within-group change compared to PRE: *P < 0.05, **P < 0.01

At PRE, CMJ height did not differ (*P* > 0.05) between VL20M (31.1 ± 4.5 cm) and VLM40 (34.1 ± 3.9 cm) nor between VL20W (24.1 ± 3.5 cm) and VL40W (23.6 ± 5.3 cm). All groups showed significant increases in CMJ from PRE to MID and all but VL40M showed significant increases from PRE to POST (Fig. 3A), but no between-group differences were observed. Effect sizes for between-group differences in relative changes PRE to POST showed a small effect in both men (*g* = 0.12) and women (*g* = 0.27).

Averaged EMG during the concentric phase of CMJ significantly increased in three of the groups (VL40M, VL20W and VL40W) at MID compared to PRE (Fig. 3B), and significance remained at POST in VL40M and VL20W. Such a pattern of increase followed by plateau/decrease was observed in both muscles separately and when averaged.

### Vastus lateralis cross-sectional area

**Fig. 4:**
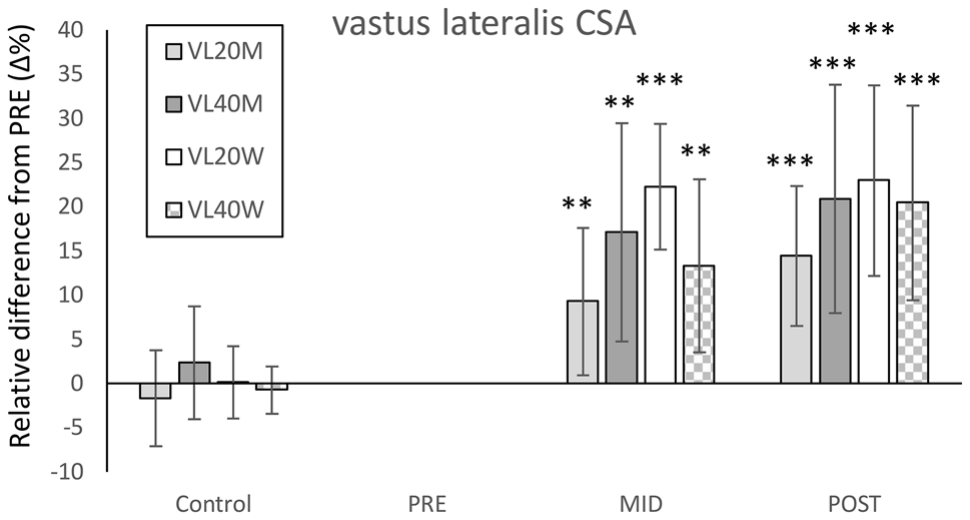
Mean (± SD) relative changes (Δ%) in vastus lateralis cross-sectional area (CSA) in all groups during the 8-week velocity-based intervention. VL20M = men training until 20% of velocity loss, VL40M = men training until 40% of velocity loss, VL20W = women training until 20% of velocity loss, VL40W = women training until 40% of velocity loss within each set. Within-group change compared to PRE: **P < 0.01, ***P < 0.001

At PRE, vastus lateralis CSA did not differ (*P* > 0.05) between VL20M (31.4 ± 3.6 cm^2^) and VL40M (31.0 ± 5.3 cm^2^) nor between VL20W (22.0 ± 4.3 cm^2^) and VL40W (21.4 ± 3.3 cm^2^). All groups increased vastus lateralis CSA, but there were no statistically significant between-group differences in training-induced increases (Fig. 4). Effect sizes for between-group differences in relative changes PRE to POST showed a medium effect (*g* = 0.59) in men and a small effect (*g* = 0.23) in women.

## Discussion

The present study showed that training to 20% or 40% of velocity loss leads to robust and similar increases in maximum strength, lifting velocity with submaximal loads, CMJ height, and vastus lateralis CSA. The results also show that recreationally trained men and women can achieve similar gains over 8 weeks (15 sessions) of training from a volume of approximately 70% in back squat and approximately 60% in bench press. Women showed very similar increases in vastus lateralis CSA throughout the training between 20 and 40% velocity-loss protocols. A medium effect size in men (21 ± 13% versus 14 ± 8%, *g* = 0.59) suggests that the typical pattern of greater gains from 40% velocity-loss training occurred, which is supported statistically by the time × sex × velocity-loss interaction (*F* = 5.89, *P* = 0.02). A unique finding of the present study is that women training to 40% velocity loss appear to benefit more (e.g., large effect sizes) for maximum strength and velocity gains with submaximal-load in bench press than training to 20% velocity loss, despite similar increases in SQ, CMJ and vastus lateralis CSA between groups.

In the present study, the same absolute (submaximal) loads were used to test maximum concentric velocity performance before and after training using an incremental test. The velocities were divided into “low” and “high” based on velocity cut-offs purported to represent 70% of 1-RM; the median relative load trained during the present intervention. VL20M and VL40M showed very similar improvements in low- and high-velocity SQ MPV and also low-velocity BP MPV, but only the VL20M group increased in high velocity in BP, matching previous findings in men (Pareja-Blanco et al. 2020a, b). On the other hand, large effect sizes in women suggest that training until 40% velocity-loss women may have additional benefit for improvements in MPV using both low and high velocities compared to 20% velocity loss (46 ± 16% vs. 26 ± 10% vs, *g* = 1.42 and 16 ± 9% vs. 7 ± 9%, *g* = 0.98, respectively). At least partly attributable for this observation could be the different (n.s.) magnitude of increase in 1-RM (17 ± 8% vs. 12 ± 8%, *g* = 0.60, for VL40W vs. VL20W, respectively). This speculation is also supported by the positive relationships observed between changes in 1-RM and MPV in both SQ and BP exercises (*r* = 0.467‒0.518, *P* < 0.05) in women.

Women may benefit from greater volume during power training from their higher fatigue-resistance, in general, compared to men (Ansdell et al. 2019). When performing the same loading protocol, women have been repeatedly shown to exhibit lower levels of acute neuromuscular fatigue than men (Häkkinen 1994; Linnamo et al. 1998). Linnamo et al. (1998) observed that 5 × 10 × 40% 1-RM leg press power loading led to lower reductions in maximal and rapid force production in women (approx. − 10%) compared to men (approx. − 25%), and recovery was almost complete 1 h after the loading in the women. Similar findings of greater acute fatigue in men have been noted following other types of resistance loading protocols (Häkkinen 1994; Taipale and Häkkinen 2013). Potential reasons for women’s lower fatigability have been proposed to be, e.g., lower muscle mass allowing greater muscle perfusion (Yoon et al. 2007) and greater type I muscle fiber content (Simoneau et al. 1985). Therefore, there may be additional benefit for women to train with a higher volume in order to improve both strength and power performance.

Statistically significant and relatively large improvements were observed in vastus lateralis CSA for all four groups. Increases of ~ 20% in the present study may be a little surprising considering that power training typically does not induce significant increases in muscle mass (Häkkinen et al. 1990). Such improvement over 15 training sessions (an average of ~ 0.32% per day) is higher than the average increases reported in the literature (Wernbom et al. 2007) and is larger than the 11‒13% increase over 10 weeks of training we observed in a previous study in trained men using the same ultrasound methods (Walker et al. 2016). This may be reflective of the subjects’ more limited experience in resistance training than, e.g., chronic resistance trainers of previous studies (e.g., Ahtiainen et al. 2003; Pareja-Blanco et al. 2017; Walker et al. 2016). However, it should be considered that VBRT requires that every repetition is performed at maximal velocity, which implies that higher force is applied in each repetition (Schilling et al. 2008) and higher activation of Type II fibers (Desmedt and Godaux 1977), along with high metabolic response (Pareja-Blanco et al. 2014). This fact may favor a positive enviroment to maximize the hypertrophic response.

The training program had a great impact on muscle mass independent of the magnitude of velocity loss per set. There are mixed findings in the literature regarding whether training closer to concentric failure will induce greater muscle hypertrophy. One possible reason for the discrepancies in velocity-loss literature is the different methods used to assess muscle mass. Pareja-Blanco et al. (2017) showed differences between 20 and 40% velocity loss via combined CSA of the vastus lateralis + intermedius using MRI while assessments of vastus lateralis using panoramic ultrasound, as in the present study, did not determine differences (Pareja-Blanco et al. 2020a). Hence, there may be reduced sensitivity to detect small differences between-groups when using one muscle and measurement cite to assess quadriceps CSA with ultrasound.

Nevertheless, our findings of similar increases in vastus lateralis CSA agree with a recent meta-analysis suggesting that training (close) to failure did not provide additional benefit (Grgic et al. 2021), and another study where rapid concentric action did not dilute hypertrophy gains versus a controlled lifting tempo (Sampson and Groeller 2016). However, training with too few repetitions per set (e.g., 10% velocity loss) would seem to compromise muscle hypertrophy gains (Pareja-Blanco et al. 2020a), at least in men. This is perhaps supported by our effect size estimations from the present study (VL20M: ~ 14% versus VL40M: ~ 21%, *g* = 0.59).

CMJ height improved significantly in all groups during training, although some fluctuations in men groups meant that improvements in VL40M was not at the level of statistical significance at POST. Based on findings by Pareja-Blanco et al. (2017), it may have been expected that 20% velocity loss would have led to preferential gains in CMJ, at least in men, but this was not observed. It seems that the between-group differences observed in the aforementioned study may be attributed to a blunted response to training in the 40% VBRT group (~ 3.5% improvement). Whereas all the groups in the present study attained similar magnitudes of improvement as the ~ 9% improvement in the Pareja-Blanco et al. (2017) 20% velocity-loss group (PRE to POST changes VL20M: ~ 7%, VL40M: ~ 6%, VL20W: ~ 8%, VL40W: ~ 10%).

Improvements in CMJ height were accompanied by increases in muscle activity as measured by surface EMG. Increased ability to activate muscle rapidly likely, at least partly, contributed to improved CMJ performance. Despite the well-known limitations in surface EMG to infer neural adaptations (Farina et al. 2014), such changes in motor unit recruitment and discharge rate have been observed following training with fast contractions (Van Cutsem et al. 1998). Therefore, it seems plausible that even training to 20% velocity loss (i.e., approx. half the training volume) is a sufficient stimulus for adaptations in muscle activation when each (concentric) repetition is performed with maximal velocity.

### Methodological considerations and potential limitations

One aspect of the study design that may have influenced the ability to determine between-group differences is that the training program lasted 8 weeks. In practice, typical power training mesocycles are 4–6 weeks in duration. It has been shown that power training for 7–12 weeks or longer can lead to a plateau or even reverse some gains in power performance both in men and women depending on the type of the training protocol and the measurements conducted (Häkkinen and Komi 1985; Kyröläinen et al. 1989; Peltonen et al. 2018). Therefore, the possibility that true peaks in performance gains being missed by the testing at weeks 5 and 10 cannot be discounted.

In standardizing the velocity loss for each group (either 20% or 40%), the present training program provides a similar training stimulus by varying the training volume according to the fatigability of the individual. Against our initial prediction, women did not perform a greater total number of repetitions compared to men; men actually performed more repetitions in BP than women. Indeed, the discrepancy of total volume between 20 and 40% velocity loss was larger for BP (~ 73% vs. ~ 62%) than for SQ (~ 78% vs. ~ 76%), and this could potentially be explored in future. Hence, it is possible that VL20W did not attain the required training volume to maximize adaptation in BP.

Despite not including a control group, the present study included a control period, which highlights the variability in the measurements and also the impact of learning. Upon inspecting the variations from Control to PRE, it is clear that the observed changes are a result of our training program and not factors external to the intervention. Therefore, the observations of improvement throughout the training period can be considered as training-induced adaptations. Nevertheless, one weakness of the present study is that three of the groups contained 11 subjects (below the a priori determined requirement of 12) at the end of the intervention; due to drop-outs. This may, at least partly, explain why statistically significant differences in women were not found despite medium and large effect sizes between-groups for bench press data. However, this was the first study conducted in women, and therefore the estimations based on previous men’s data may not be appropriate. Hence, the present study provides a guide for future VBRT studies investigating adaptations in women.

## Conclusion

Robust and similar increases in strength and power performance were observed in men and women over 8 weeks of VBRT regardless of whether training until 20% or 40% velocity loss. Performance increases were accompanied by increased quadriceps muscle activity and cross-sectional area with no statistically significant differences between-groups. Conversely, subtle but potentially meaningful greater gains in strength and lifting velocity were observed following 40% velocity-loss training in women, which was absent in comparison between men groups. It may be that women require a greater velocity loss (i.e., within-set fatigue) than men, especially in bench press, to maximize strength and power development. It, therefore, appears that programming of power training in women should consist of higher volume than currently used to induce adaptations in men.

## Abbreviations

SQ: (Full) Back squat
BP: Bench press
CMJ: Countermovement jump
CSA: Cross-sectional area
MPV: Mean propulsive velocity
MPP: Mean propulsive power
1-RM: One-repetition maximum
EMG: Surface electromyography
VBRT: Velocity-based resistance training
